# Welfare Conditions of Donkeys in Europe: Initial Outcomes from On-Farm Assessment

**DOI:** 10.3390/ani6010005

**Published:** 2016-01-08

**Authors:** Francesca Dai, Emanuela Dalla Costa, Leigh Margareth Anne Murray, Elisabetta Canali, Michela Minero

**Affiliations:** Dipartimento di Scienze Veterinarie e Sanità Pubblica, Università degli Studi di Milano, via Celoria 10, 20133 Milano, Italy; emanuela.dallacosta@unimi.it (E.D.C.); leigh_murray75@hotmail.co.uk (L.M.A.M.); elisabetta.canali@unimi.it (E.C.); michela.minero@unimi.it (M.M.)

**Keywords:** animal-based indicators, animal welfare, AWIN, equine, donkey

## Abstract

**Simple Summary:**

This paper aims to present the first outcomes of data collected using the AWIN welfare assessment protocol for donkeys in 20 EU donkey facilities. Three assessors evaluated 278 donkeys. The authors found recurrent issues: tendency to obesity, lack of hoof care and irregular positive interactions with humans. The protocol proved to be applicable in different management conditions and for donkeys of different attitude.

**Abstract:**

This paper is a baseline study to present the initial outcomes of data collected in a sample of EU donkey farms using the AWIN welfare assessment protocol for donkeys, comprehensive of 22 valid, reliable and feasible animal-based indicators. A total of 20 donkey facilities (N = 12 in Italy and N = 8 in United Kingdom) were visited and 278 donkeys of different breed, aged 2–45 years, were assessed. Three assessors underwent a common training period to learn how to perform and score all the indicators included in the protocol. Data was collected using digitalized systems and downloaded to a database. A descriptive statistic for each welfare indicator was calculated. The authors found recurrent issues: 25% of donkeys were moderately over weight; although most of the assessed animals had good quality hoof care, 15.16% of them presented some signs of neglect, such as overgrowth and/or incorrect trimming; 18.05% of donkeys showed an avoidance reaction to an approaching human in the avoidance distance test. The protocol has proven to be applicable in different management conditions and for donkeys of different attitude.

## 1. Introduction

Worldwide, it estimated that there are approximately 43 million donkeys [1]; most of them contribute directly and indirectly to people’s livelihoods in developing countries, where they are used as draught animals and/or in agriculture [2,3,4]. Across Europe, the purpose of keeping donkeys is constantly changing. While they are still used as working animals in some parts of the continent, elsewhere they are kept as pets, or used for leisure activities, therapy programs, or milk and meat production. As donkeys are employed for various activities, their management can differ considerably and they can easily become invisible to official national databases and figures; there are challenges in getting accurate population statistics for donkeys and in assessing their economic value to a country. Hence, it is not surprising that objective information about donkey welfare conditions is limited or fragmented. To date, limited information regarding welfare assessment and recommendations of donkeys in EU is available in the literature [5,6,7], whilst most of the scientific papers report results for welfare assessment of donkeys in developing countries [2,3,4,8,9,10,11,12]. The AWIN (Animal Welfare Indicators) project, funded by the European Commission in the Seventh Framework Programme, aimed to improve welfare of several species, including donkeys, by developing scientifically sound and practical on-farm welfare assessment protocols. In 2015, following the same method used for other species [13,14], AWIN researchers published the welfare assessment protocol for donkeys [15], including 22 valid, reliable and feasible animal-based indicators. The protocol is intended to function as a welfare assessment tool for donkeys over one year old and can be downloaded freely from http://dx.doi.org/10.13130/AWIN_DONKEYS_2015. This paper aims to present the first outcomes of data collected in a sample of EU donkey facilities using the AWIN welfare assessment protocol for donkeys.

## 2. Experimental Section

### 2.1. Facilities and Animals

A total of 20 donkey facilities (N = 12 in Italy and N = 8 in United Kingdom) were visited between March and June 2014. A representative sample of donkey facilities was selected using a quota sampling method according to their geographical distribution (Northern, Central and Southern Country) and animal attitude (35% of farms were rescue centres, 25% dairy farms, 25% used donkeys for Assisted Therapies, 10% riding centres and 5% zoo). All the donkey owners were contacted over the phone and participated in the study on a voluntary basis. The number of donkeys per facility ranged from three to 394 (mean = 75.50). A total of 278 donkeys (females = 140, pregnant females = 29, geldings = 94, stallions = 13), of different breeds and aged 2–45 years (mean = 12.50) were assessed. In each facility, the number of donkeys to be assessed was determined according to the table reported in the protocol [15], which is calculated for an expected variation in data of 0.5, at the level of confidence of 0.9 and a precision of the estimate (δ) of 0.1. Random selection of donkeys was performed in order to prevent the many possible sources of bias that could affect animal sampling on-farm.

### 2.2. Assessors

Three female experimenters (aged 30–37 years), two veterinarians and one animal scientist, were selected to perform the assessments. The assessors already had a good knowledge of donkey behaviour and welfare. Before carrying out the on-farm assessment, they underwent a common training period to learn how to perform and score all the indicators included in the protocol. The training of assessors consisted of two phases: first e-learning and then face-to-face. Each welfare indicator was transferred into an e-learning object organized in different sections: description, how to assess, how to score, examples and self-assessment exercises. The face-to-face training, lasting one day and consisting of theoretical and practical on-farm training, aimed to teach the practical skills necessary to perform and score all the indicators accurately and reliably. Experimenters independently assessed twelve donkeys, kept as pets in a farmhouse. Both phases ended with an assessment of learning; the training was considered complete when the assessors achieved ≥80% of correct responses and ≥80% agreement with the silver standard, a senior researcher of the AWIN project, with over 10 years’ experience in assessing equine welfare.

### 2.3. Welfare Assessment

The assessment was conducted using the AWIN welfare assessment protocol for donkeys (Table 1), available at http://dx.doi.org/10.13130/AWIN_DONKEYS_2015. The protocol offers a two-level assessment. The first level is a quick screening, consisting of a selection of 20 robust and feasible indicators, which cover all the principles developed by Welfare Quality^®^, can be readily applied and require minimal handling of animals. Time needed approximately for assessing a donkey is 5 min. Most indicators are animal-based; however, when animal-based indicators responding to the required characteristics were not available, or could not be developed in AWIN, resource- or management-based measures are used in order to assess a given welfare aspect (e.g., evaluation of bedding and shelter dimension for “comfort around resting”). The second-level welfare assessment, a more comprehensive and in-depth assessment, was adopted for all facilities included in the sample. According to the protocol indications, the second level is recommended where there is only one donkey at the premises or when a noncompliance with the current legislation is pointed out or, as well, when the within-farm proportion of animals meeting a given criterion is lower than the proportion of animals observed in the worst 5% of the farms of the reference population. Twenty-two indicators are included in the second level assessment. Twenty of them are assessed following the same procedure adopted in the first level, whilst the Qualitative Behaviour Assessment and Skin Tent Test are conducted only in the second level. The approximate time needed to assess a donkey is 10 min.

**Table 1:** The AWIN indicators for donkeys, for different welfare principles and criteria.

Welfare Principles	Welfare Criteria	Welfare Indicators
Good Feeding	Appropriate nutrition	Body Condition Score
	Absence of prolonged thirst	Skin tent test
		Water availability
Good Housing	Comfort around resting	Bedding
		Shelter dimensions
	Thermal comfort	Signs of thermal stress
	Ease of movement	Not considered relevant to extensive animals
Good Health	Absence of injuries	Integument alterations
		Swollen joints
		Lameness
		Prolapse
	Absence of disease	Hair coat condition
		Faecal soiling
		Discharges
		Cheek palpation
		Abnormal breathing
		Coughing
	Absence of pain induced by management procedures	Signs of hoof neglect
		Signs of hot branding
Appropriate Behaviour	Expression of social behaviour	Social interaction
	Expression of other behaviours	Stereotypies
	Good human–animal relationship	Human–animal relationship tests
	Positive emotional state	Qualitative Behaviour Assessment

### 2.4. Data Collection and Data Analysis

Data was collected using Open Data Kit application (developed by University of Washington, Department of Computer Science and Engineering), a digitalized system available for Android devices (for further information see [16]). As for QBA, the assessors used a dedicated electronic Android application, specifically developed for QBA automated data recording and analysis. Data was downloaded from the server to a Microsoft Excel file (Microsoft Corporation 2010) and then analysed with the SPPS statistical package (IBM SPSS Statistic 21). The proportion of donkeys with different scores for each welfare indicator was calculated. QBA scores were automatically downloaded from the QBA App to an Excel file. To analyse QBA scores, a Principal Component Analysis (PCA, correlation matrix, no rotation) was conducted.

## 3. Results and Discussion

### 3.1. Prevalence of Welfare Issues

#### 3.1.1. Good Feeding

Results regarding indicators for “good feeding” are reported in the Table 2. Most of the donkeys (63%) showed a good body condition score (BCS = 3), a consequence of appropriate nutrition. Extremes were rare (0.36% and 1.08%, for BCS 1 and 5 respectively). It is remarkable that 25% of the assessed donkeys were moderately overweight, with a body condition score of 4. The possible explanation is that donkeys are very efficient at metabolising feed, therefore their energy requirements are lower than a similar sized pony [17]; this can result in overfeeding and, in the long term, obesity. Our results are in contrast with outcomes of researches in developing countries: working donkeys tend more often to be lean rather than fat [2,12,18]. In European Countries, obesity is a serious and increasing cause of concern in donkeys, as it puts them at risk of developing serious and potentially fatal diseases, such as hyperlipemia, laminitis and Cushing's Syndrome [6,19,20]. For this reason, educating owners about the nutrition requirements of donkeys and about feeding practices (e.g., limiting availability of feed or pasture time to control for obesity) is of primary importance; it is also relevant to make them aware of the possible life-threatening conditions that arise and teach them to regularly check the body condition score of their animals.

**Table 2:** Results of “good feeding”.

Welfare Indicator	%
Good Feeding	
**BCS**	
1	0.36
2	6.85
3	63.17
4	25.27
5	1.08
NA	3.25
**Skin tent test**	
Loss of elasticity	0.72
No loss of elasticity	98.19
NA	1.08
**Water point**	
Automatic drinker	65
Bucket	35
No	0
**Water point cleanliness**	
Clean	65
Dirty	15
Very dirty	20
**Water point functioning**	
Yes	65
No	0
NA	35

Only two donkeys presented a loss of skin elasticity in the skin tent test. This test is considered as indicative of a dehydration status, while previous studies reported contradictory results and suggested that the response to the skin tent test can be influenced by other factors (e.g., age and thinness) [11,21,22]. Capillary refill time is generally used to assess tissue perfusion and cardiovascular performance [23], but it cannot be considered a suitable alternative indicator of absence of thirst as it was proven not to be specific for dehydration [24]. All the facilities visited were equipped with water points (65% automatic drinkers, 35% buckets), although sometimes they were dirty or very dirty, 15% and 20%, respectively. Dirtiness of water is a recognized cause of reluctance to drink in donkeys [19]; certainly, contaminants such as faeces and mould can have an impact on the appearance, odour, and taste of water. The first step in improving cleanliness of water points is to educate owners about good watering practices, including regular cleaning of drinkers and buckets.

#### 3.1.2. Good Housing

No donkeys presented any signs of thermal stress, neither hot nor cold; this was probably due to the fact that no extreme weather conditions were registered during the assessments (Table 3). Assessing signs of thermal stress is regardless an important part of welfare assessment, as donkeys may suffer during hot periods and they do not have the same waterproof coat as horses, making them vulnerable to heavy rain, snow, hail or strong winds.

Ninety percent of facilities had pastures or paddocks for the donkeys; only one farm had just an outdoor area with concrete floor. Eighty-five percent of facilities provided shelters of appropriate size, while it was noteworthy that 15% had no shelter at all. Forty-five percent of facilities did not provide any bedding material to donkeys which had permanent access to pasture. Clean bedding was used in the remaining 55% of facilities and only in one facility was the bedding quantity considered insufficient (floor areas clearly visible).

**Table 3:** Results of “good housing”.

Welfare Indicator	%
Good Housing	
**Signs of thermal stress**	
Present	0
Absent	100
NA	0
**Shelter**	
Yes	85
No	15
**Bedding**	
No	45
Yes	55
**Bedding quantity**	
Sufficient	45
Insufficient	5
NA	50
**Bedding cleanliness**	
Clean	50
Dirty	0
NA	50

#### 3.1.3. Good Health

As shown in Table 4, more than 80% of the assessed donkeys had no swollen joints, lameness, unhealthy coat, discharges, faecal soiling, dyspnoea, signs of hot branding, and signs of teeth abnormalities. It is worth mentioning that local alterations in coat condition, changing coat, breed characteristics, alterations to the coat caused by harnessing were not considered. The prevalence of integument alterations was 31.05%: this indicator includes hairless patches, superficial skin lesions and deep wounds. Most of the integument alterations were hairless patches (25.99%) that could be caused by parasites, mycosis or other diseases causing itching. Superficial skin lesions were observed in 8.66% of animals and they were due to small traumas like bites from other donkeys or collision with protruding and sharp-edged parts of housing structures, while deep wounds were rare, with only 0.72%. It is important to underline that all the superficial skin lesions and deep wounds were medicated, pointing to the fact that owners were generally committed to protecting their donkeys′ health.

Good hoof care is essential to minimize incidence of foot problems [25,26]. Although most of the assessed donkeys had good quality hoof care, 15.16% of the animals presented some signs of neglect, such as overgrowth and/or incorrect trimming. Incorrect management of the donkey′s foot could cause lameness and chronic foot disease, which could be painful for the donkey [25,26].

**Table 4:** Results of “good health”.

Welfare Indicator	%
Good Health	
**Integument alterations**	
Present	31.05
Absent	68.95
**Swollen joints**	
Present	1.08
Absent	98.92
**Lameness**	
Lame	1.81
Not lame	98.19
**Hair coat condition**	
Unhealthy	10.47
Healthy	89.53
**Ocular discharge**	
Present	16.25
Absent	83.75
**Nasal discharges**	
Present	0.72
Absent	99.28
**Faecal soiling**	
Present	2.53
Absent	97.47
**Abnormal breathing**	
Present	0.36
Absent	99.64
**Signs of hot branding**	
Present	0.36
Absent	99.64
**Signs of hoof neglect**	
Present	15.16
Absent	84.84
**Cheek palpation**	
Presence of abnormalities	2.17
No abnormalities	90.25
NA	7.58

#### 3.1.4. Appropriate Behaviour

Results regarding the principle “appropriate behaviour” are reported in Table 5. Most of the assessed donkeys (>70%) showed no signs of stereotypies and a positive relationship with humans. To date, the literature does not report any specific studies on stereotypies in donkeys; however, Cox *et al.* stated that it is possible to observe them [6]. We found one stallion, kept alone, showing weaving and box walking and one jenny, kept in a paddock with other donkeys, head nodding [27]. Comparative studies of stereotypic behaviour in donkeys and horses could prove useful to provide a better understanding of the basis of these behavioural problems in both species.

**Table 5:** Results of “appropriate behaviour”.

Welfare Indicator	%
Appropriate Behaviour	
**Stereotypies**	
Evidence of stereotypies	0.72
No evidence of stereotypies	99.28
**Avoidance Distance**	
Avoidance behaviour	18.05
No avoidance	78.70
NA	3.25
**Walking Down Side**	
Negative signs	8.66
Neutral/Positive signs	88.45
NA	2.89
**Tail tuck**	
Present	11.55
Absent	86.28
NA	2.17
**Social Interactions**	
No social contact	0
Social contact	100

The human–animal relationship, evaluated using the human–animal behaviour tests described by Dalla Costa and colleagues in 2015 [28] (Avoidance Distance test, Walking Down Side test and presence of tail tuck), was generally positive, with more than 70% of donkeys showing a positive reaction when approached by an unknown person and no signs of fear (absence of tail tuck). These results apparently differ from the findings of previous studies, conducted on working donkeys: Pritchard *et al.* reported that 44.30% of donkeys had a negative reaction towards an observer performing an approach test and 28% responded to Walking Down Side test by clamping down the tail and tucking in the hindquarters [2]. Burn *et al.* found that 25.90% of donkeys showed an avoidance reaction to an approaching observer and 21.20% performed tail tuck [9]. The comparison with the above mentioned studies, however, should be considered with due caution as donkeys in developing countries might be subjected to different types of stressors such as long work hours or poor nutrition. Other studies performed in a more comparable context [6,7] did not evaluate direct animal-based measures and therefore it is not clear to what extent their outcomes can be linked to the present findings. In our research, 18.05% of donkeys showed an avoidance reaction. One likely explanation for this result is that they were not used to being approached by people when restrained or they negatively associated the connection between handling and the presence of veterinarians and/or farriers performing aversive procedures. Interestingly, once in proximity of the experimenter (Walking Down Side test), most of the donkeys did not show any aggressive reaction.

All the assessed donkeys had the possibility to relate with conspecifics: they were housed in groups or, when housed singly, they had the opportunity to visually and physically interact with other donkeys. This result highlights a positive area for their welfare considering that donkeys are a highly social species and having contact with conspecifics represents an important behavioural need.

**Figure 1:**
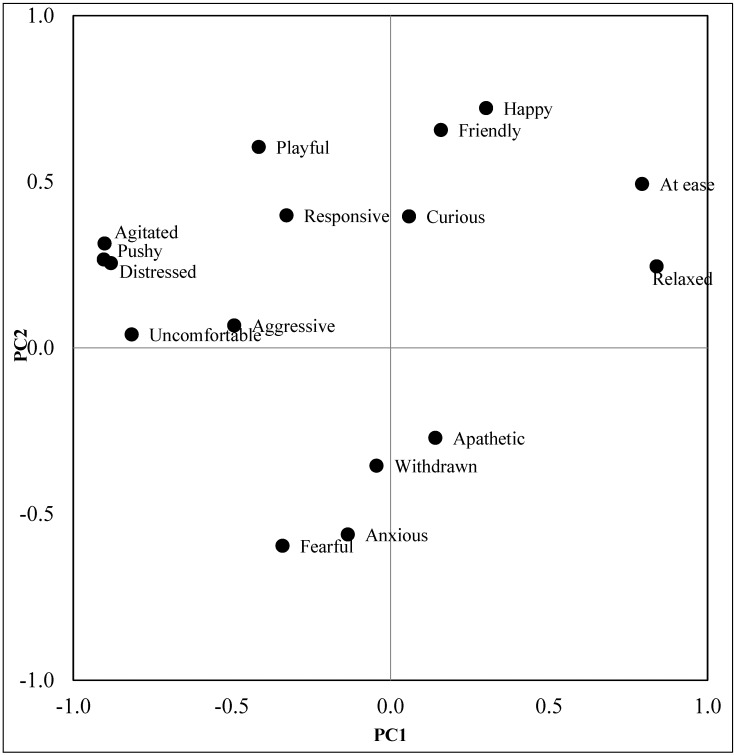
Loading plot of the descriptors on the first and second Principal Components (PC1 and PC2).

In order to assess positive emotional states, the AWIN Welfare Assessment Protocol for Donkeys includes, as part of the second level assessment, the Qualitative Behaviour Assessment (QBA). This method relies on the ability of humans to integrate perceived details of behaviour, and the assessment was performed following methods described by Minero and colleagues [29]. The PCA of the QBA assessments for the 20 facilities visited revealed five main factors with Eigen values greater than 1; the first three components together account for 67.31% of variation between donkey facilities. Figure 1 shows the distribution of the descriptors along the first two PCA factors. Many of the terms load on the first Principal Component, accounting for 32.56% of the total variance and range from at ease/relaxed to agitated/distressed, which suggests that this component is important in the description of the level of arousal of donkeys. Component 2 counts for 19.05% of variance and seems to be more related to the valence of donkeys′ affective states, ranging from happy/friendly to anxious/fearful. Animals with high positive scores on this component were described as being in a more positive emotional state than donkeys with high negative scores.

#### 3.1.5. From First- to Second-Level Welfare Assessment

According to the indications described in the AWIN welfare assessment protocol for donkeys, three out of 20 facilities should have required a second-level welfare assessment. One facility did not meet the criterion of absence of disease, with only 33% of donkeys free from discharges, bad coat condition or signs of diarrhoea. One facility did not meet the criterion of absence of pain, with only one donkey out of six exhibiting good foot conditions. The remaining facility did not meet the criterion appropriate nutrition, since all the donkeys had a BCS = 4.

### 3.2. Feasibility and Acceptability

In Italy, all the donkey owners contacted allowed researchers to enter their facilities. In the UK, seven owners declared that they were not interested in taking part in the study and therefore their facilities were replaced by other facilities of the same herd size. All the stable managers allowed the assessors to enter the stables/paddocks and approach the donkeys and were generally interested and collaborative.

It was not always possible to collect some indicators: 3.25% of donkeys were not assessed for Body Condition Score, 1.08% for the Skin Tent test, 7.58% for dental abnormalities (cheek palpation), 3.25% for Avoidance Distance, 2.89% for Walking Down Side and 2.17% for presence of tail tuck. The prevailing reason for not being able to assess these indicators was that, in some facilities, donkeys were not used to being restrained and tried to escape from the unknown experimenters. In these cases, handling donkeys with a head-collar and a rope was not possible. Since routine procedures such as veterinary and farrier checks require the donkeys′ restraint, a high prevalence of donkeys which are difficult to restrain is *per se* informative of likely aversive human–animal interactions during routine procedures.

## 4. Conclusions

The number of assessed facilities was relatively small, due to budget and time constraints; however, the applied protocol has proven to be applicable in different management conditions and for donkeys of different attitudes. The authors consistently pointed out issues such as tendency to obesity, lack of hoof care and irregular positive interactions with humans.

Feasibility and owners acceptability were good, but some concerns remain regarding restraining the donkeys; educating the owners about the importance of proper and frequent handling of animals could improve both the human–animal relationship and feasibility of the protocol.
